# *TP63* Transcripts Play Opposite Roles in Chicken Skeletal Muscle Differentiation

**DOI:** 10.3389/fphys.2018.01298

**Published:** 2018-09-18

**Authors:** Wen Luo, Xueyi Ren, Jiahui Chen, Limin Li, Shiyi Lu, Tian Chen, Qinghua Nie, Xiquan Zhang

**Affiliations:** ^1^Department of Animal Genetics, Breeding and Reproduction, College of Animal Science, South China Agricultural University, Guangzhou, China; ^2^Guangdong Provincial Key Lab of Agro-Animal Genomics and Molecular Breeding, Key Lab of Chicken Genetics, Breeding and Reproduction, Ministry of Agriculture, South China Agricultural University, Guangzhou, China

**Keywords:** TAp63α, ΔNp63α, chicken, myoblast differentiation, cell cycle

## Abstract

Tumor protein 63 (TP63) comprises multiple isoforms and plays an important role during embryonic development. It has been shown that *TP63* knockdown inhibits myogenic differentiation, but which isoform is involved in the underlying myogenic regulation remains uncertain. Here, we found that two transcripts of *TP63*, namely, *TAp63*α and Δ*Np63*α, are expressed in chicken skeletal muscle. These two transcripts have distinct expression patterns and opposite functions in skeletal muscle development. *TAp63* has higher expression in skeletal muscle than in other tissues, and its expression is gradually upregulated during chicken primary myoblast differentiation. Δ*Np63* can be expressed in multiple tissues and exhibits stable expression during myoblast differentiation. *TAp63*α overexpression inhibits myoblast proliferation, induces cell cycle arrest, and enhances myoblast differentiation. However, although Δ*Np63*α has no significant effect on cell proliferation, the overexpression of Δ*Np63*α inhibits myoblast differentiation. Using isoform-specific overexpression assays following RNA-sequencing, we identified potential downstream genes of *TAp63*α and Δ*Np63*α in myoblast. Bioinformatics analyses and experimental verification results showed that the differentially expressed genes (DEGs) between the *TAp63*α and control groups were enriched in the cell cycle pathway, whereas the DEGs between the Δ*Np63*α and control groups were enriched in muscle system process, muscle contraction, and myopathy. These findings provide new insights into the function and expression of *TP63* during skeletal muscle development, and indicate that one gene may play two opposite roles during a single cellular process.

## Introduction

Tumor protein 63 is a p53 family member required for limb, craniofacial and epithelial development (Yang et al., 1999). Unlike for *p53*, several messenger RNAs are transcribed from the *TP63* gene due to the use of two promoters and to alternative splicing (Lin et al., 2015). These mRNAs encode at least six TP63 isoforms (Guo and Mills, 2007). Isoforms with the N-terminal transactivation (TA) domain are referred to as the TA isoforms, and the N-terminal truncated (ΔN) isoform lacks the TA domain. It is well known that TP63 is involved in the formation of the epidermis. However, different TP63 isoforms perform different functions during epithelial development. ΔNp63 isoforms are important for maintaining the proliferative potential of the basal layer, whereas TAp63 isoforms contribute to late stage differentiation in mature keratinocytes (Candi et al., 2007). Different TP63 isoforms probably regulate gene sets that have completely distinct biological functions (Wu et al., 2003), and different isoforms may perform cell-type specific functions (Guo and Mills, 2007). For example, TAp63α promotes proliferation in the mouse epidermis (Koster et al., 2006), while it induces apoptosis in Hep3B cells (Gressner et al., 2005). ΔNp63 overexpression promotes HNSCC cell survival (Rocco et al., 2006), while it induces apoptosis in the non-small cell lung carcinoma cell line H1299 (Lo et al., 2006). Therefore, it is important to distinguish the different functions of TP63 isoforms during different cellular processes.

Skeletal muscle development is a complex process that is regulated at multiple levels. Many transcription factors and miRNAs are involved in the regulation of myogenesis (Braun and Gautel, 2011; Luo et al., 2013). It has been shown that p53 family members play a role in controlling myogenic differentiation (Cam et al., 2006). The p53 protein transactivates the *RB* gene, which plays a critical role in cell cycle exit in differentiated myocytes (Novitch et al., 1996). p63 and p73 induce the transcription of p57, maintain RB protein activity, and facilitate myogenic differentiation (Cam et al., 2006). However, the isoforms of TP63 have never been addressed in these studies. Which isoform is expressed during myogenic differentiation, and which isoform plays a major role in myogenic differentiation remain unclear. Recently, it was found that one of the TP63 isoforms, TAp63gamma, is involved in myogenic differentiation, and that the knockdown of TAp63 inhibited myotube formation (Cefalu et al., 2015). However, there are no results describing the expression and function of any other TP63 isoforms.

miR-203 is widely known as a skin-specific miRNA that plays an important role in epidermal development (Yi et al., 2008). miR-203 can regulate epidermal stratification and differentiation by directly repressing the expression of TP63 (Lena et al., 2008). However, in our previous work, we found that the “skin-specific miRNA” miR-203 could also be expressed in and function in the development of skeletal muscle (Luo et al., 2014). During muscle differentiation, miR-203 inhibits myoblast proliferation and differentiation by repressing *c-Jun* and *MEF2C*, respectively. In addition to *c-Jun* and *MEF2C*, *TP63* was also found to be a direct target gene of miR-203 in skeletal muscle. Considering that *TP63* has diverse transcripts and plays roles in muscle development in mammals, here, we explored its transcription, expression, and functional significance in chicken myoblast proliferation and differentiation. These results were important for understanding the function and regulation of *TP63* isoforms in myogenesis.

## Materials and Methods

### Ethics Statement

This study was carried out in accordance with the principles of the Basel Declaration and recommendations of the Statute on the Administration of Laboratory Animal, the South China Agriculture University Institutional Animal Care and Use Committee. The protocol was approved by the South China Agriculture University Institutional Animal Care and Use Committee (approval ID: 2017046).

### Animals

The embryonic and 7-week-old Xinghua female chickens were used in this study. For qPCR of TP63 in different tissues, the tissues were isolated from four 7-week-old Xinghua female chickens. For primary myoblast isolation, at least six embryos at embryo day 11 (E11) were used in each experiment. The sex of each embryos was determined by PCR with the sex-specific primers (Li et al., 2017).

### Cell Culture

Chicken embryo fibroblast cell line was cultured in high-glucose Dulbecco’s modified Eagle’s medium (Gibco) with 10% fetal bovine serum and 0.2% penicillin/streptomycin. The isolation and culture of chicken primary myoblasts were carried out as previously described (Li et al., 2017).

### RNA Extraction, cDNA Synthesis, and Quantitative Real-Time PCR

Total RNA was extracted from tissues or cells using RNAiso reagent (Takara, Otsu, Japan). The reverse transcription reaction for mRNA was performed with PrimeScript RT reagent Kit with gDNA Eraser (Takara) according to manufacturer’s manual. qPCR program was carried out in Bio-Rad CFX96 Real-Time Detection System (Bio-Rad, Hercules, CA, United States) with iTaq^TM^ Universal SYBR^®^ Green Supermix (Bio-Rad). All reactions were run in triplicate. The 2^-ΔΔC_t_^ method was used to measure gene expression with *β-actin* as the reference gene (Kenneth and Thomas, 2001).

### The 5′ and 3′ Rapid Amplification of cDNA Ends (RACE)

For 5′ RACE and 3′ RACE, total RNA isolated from chicken skeletal muscle and pooled total RNAs from different tissues were used. The detailed procedure was carried out according to previously described (Luo et al., 2015). All of the primers used in RACE were summarized in **Supplementary Table S1**.

### RNA Sequencing

The chicken primary myoblasts transfected with TAp63α, ΔNp63α, or GFP control overexpression vectors were harvested and total RNA was extracted using RNAiso reagent (Takara). Then the RNA samples were sent to Beijing Genomics Institute for RNA sequencing by using BGISEQ-500 (BGI, Wuhan, China). All the sequence data have been deposited in NCBI’s Gene Expression Omnibus (GEO^1^) and are accessible through GEO series accession number GSE114452.

### Luciferase Reporter Assays

Based on the *TP63* mRNA sequence we obtained, primers for amplifying the *TP63* 3′ UTR region with predicted gga-miR-203 binding site were designed (**Supplementary Table S1**). The plasmid pmirGLO-TP63-3′UTR (wild-type) and pmirGLO-TP63-3′UTR-mutant (mutant with gga-miR-203 potential binding site deleted) were prepared for verification of target relationship between gga-miR-203 and *TP63* mRNA. gga-miR-203 mimic (50 nM, RiboBio, Guangzhou, China) and pmirGLO-TP63-3′UTR (200 ng) were co-transfected into DF-1 cells (3 × 10^4^ cells) by using Lipofectamine 3000 reagent (Invitrogen) according to the manufacturer’s instructions. After 48 h, Luc-pair Duo-Luciferase Assay Kit 2.0 (GeneCopoeia, Rockville, MD, United States) was used to analyze the activities of luciferases. The luminescent signal was quantified using Synergy 2 Multi-mode Microplate Reader (Biotek, Winooski, VT, United States) and analyzed with Gene5 software (Biotek).

### Plasmid Construction

The TP63 overexpression vectors were constructed according to the user manual of Easy Ligation Kit (Sidansai, Shanghai, China). TAp63α and ΔNp63α coding sequences were amplified from chicken leg muscle cDNA by PCR. The PCR products were cloned into the pSDS-204 vector (Sidansai). The successful TAp63α and ΔNp63α overexpression vectors were confirmed by agarose gel electrophoresis and DNA sequencing.

### Immunoblotting and Immunofluorescence

Immunoblotting and immunofluorescence were performed as previously described (Luo et al., 2016). The following antibodies were used for immunoblotting: anti-MYOG (Biorbyt, Cambridge, United Kingdom), anti-MYOD (BD Biosciences, San Jose, CA, United States), anti-MyHC (Developmental Studies Hybridoma Bank, Iowa City, IA, United States) and anti-Tubulin (Bioworld, Minneapolis, MN, United States). The protein expression were presented as the ratio between indicated protein gray value and Tubulin gray value. We set the mean expression value of pSDS204-GFP group or si-NC group to 1, and the other group was a fold change comparing to the control group. Results are mean ± SEM from three independent experiments. The following antibody and reagent were used for immunofluorescence: anti-MyHC (DSHB), FITC-conjugated anti-mouse IgG (EarthOx, Millbrae, CA, United States), 4′6-diamidino-2-phenylindole (DAPI, Beyotime, Jiangsu, China).

### Cell Cycle Analysis

After 48 h transfection of gene overexpression vectors, chicken primary myoblasts were collected and fixed in 75% ethanol overnight at -20°C. After ethanol fixation, the cells were stained with 50 μg/mL propidium iodide (Sigma) containing 10 μg/mL RNase A (Takara) and 0.2% (v/v) Triton X-100 (Sigma) for 30 min at 4°C. BD Accuri C6 flow cytometer (BD Biosciences) was subsequently used to analyze the cell cycle with Cell Cycle Analysis Kit (Thermo Fisher Scientific, Waltham, MA, United States), and the data analysis was performed using FlowJo 7.6 software (Verity Software House).

### CCK-8 Assay

Primary myoblast were cultured in 96-well plates. A total of 10 μL of Cell counting kit-8 reagent (Dojindo, Kumamoto, Japan) was added into each well and incubated for 1 h. The assay was repeated at different time points of 12, 24, 36, 48 h after transfection. The absorbance was measured at 450 nm by a Model 680 Microplate Reader (Bio-Rad). All the data were acquired by averaging the results from six independent experiments.

### RNA Oligonucleotides

Isoform-specific siRNAs against chicken *TAp63*α and Δ*Np63*α were all purchased from RiboBio (RiboBio, China). Target sequence of si-TAp63α is 5′-GGGACTTCCTGGAACAGCCAATATG-3′. Target sequence of si-ΔNp63α is 5′-CCGAGTCCTGTTATCTTCCAAGTAG-3′.

### Statistical Analysis

All data shown are mean ± SEM with at least three samples or cultures per group and three wells per culture. Well was considered the experimental unit for cell culture applications. We performed statistical analysis by using independent sample *t*-test through SPSS. We considered *p* < 0.05 to be statistically significant. ^∗^*p* < 0.05; ^∗∗^*p* < 0.01.

## Results

### *TP63* Is a gga-miR-203 Target Gene and Is Involved in Myogenic Differentiation

**FIGURE 1:**
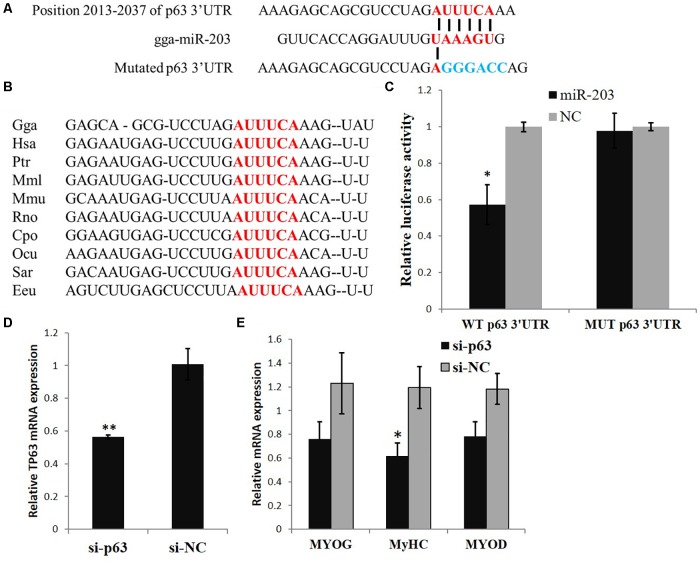
*TP63* is a gga-miR-203 target gene and is involved in myogenic differentiation. **(A)** Predicted binding site and mutated site (blue) of gga-miR-203 in the 3′ UTR of *TP63*. **(B)** The predicted binding site of gga-miR-203 in the 3′UTR of *TP63* is highly conserved among vertebrates. **(C)** Luciferase reporters were transfected into DF-1 cells with the gga-miR-203 mimic or negative control (NC) duplexes, and luciferase activity was determined 36 h after transfection. **(D)** TP63 specific siRNA transfection significantly inhibited *TP63* mRNA expression in myoblast. **(E)** The expression of muscle differentiation marker genes in myoblast after the transfection of TP63-specific siRNA. The values are presented as the mean ± SEM (*n* = 3). An independent samples *t*-test was used to analyze the statistical differences between groups. ^∗^*p* < 0.05; ^∗∗^*p* < 0.01.

In our previous study (Luo et al., 2014), we found that the expression of *TP63* was significantly downregulated when we transfected a gga-miR-203 mimic into chicken primary myoblasts. TargetScan (release 5.2) online software predicted that the *TP63* mRNA is a direct target of gga-miR-203 (**Figure 1A**), and the predicted target site of gga-miR-203 in the 3′UTR of *TP63* mRNA is highly conserved among vertebrates (**Figure 1B**). The dual-luciferase reporter gene assay confirmed that gga-miR-203 can directly bind to the predicted target site of gga-miR-203 in the 3′UTR of *TP63* mRNA (**Figure 1C**). Considering that gga-miR-203 is a negative regulator of myogenic differentiation and that *TP63* has been reported to play roles in myogenic differentiation, we next studied the roles of TP63 in chicken skeletal muscle differentiation. We synthesized a *TP63* specific siRNA and found that this siRNA can efficiently inhibit *TP63* expression (**Figure 1D**). Notably, TP63 knockdown significantly reduced the expression of *MyHC* (**Figure 1E**), which is a terminal myogenic differentiation marker gene. Therefore, these results suggested that *TP63* is a gga-miR-203 target gene and is involved in myogenic differentiation.

### *TAp63*α and Δ*Np63*α Are Two Conserved Transcripts Expressed in Chicken Tissues

**FIGURE 2:**
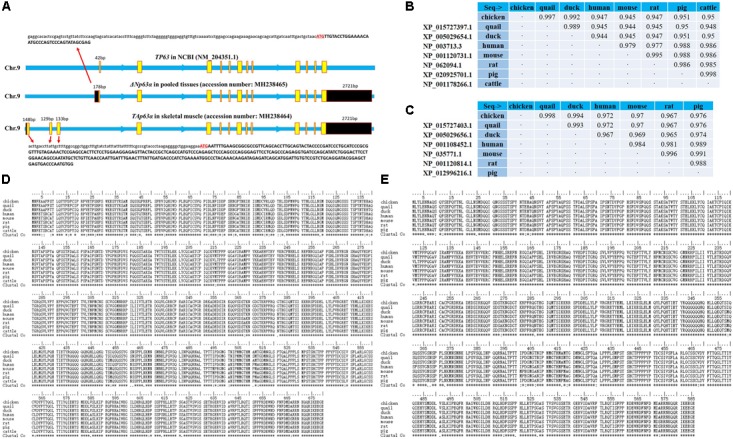
*TAp63*α and Δ*Np63*α are two conserved transcripts expressed in chicken tissues. **(A)** Genomic structure of the chicken *TP63* gene. The yellow boxes indicate coding sequence regions, and the black boxes indicate the UTRs. The capital letters indicate coding sequences, and the lowercase letters indicate UTR sequences. **(B)** Percent identities of the chicken, quail, duck, human, mouse, rat, pig, and cow TAp63α amino acid sequences. **(C)** Percent identities of the chicken, quail, duck, human, mouse, rat, and pig ΔNp63α amino acid sequences. **(D)** Amino acid alignment of TAp63α proteins from chickens, quails, ducks, humans, mice, rats, pigs, and cattle. Conserved sequences are marked with asterisk within the Clustal Co sequences. **(E)** Amino acid alignment of ΔNp63α proteins from chickens, quails, ducks, humans, mice, rats, and pigs. Conserved sequences are marked with asterisks within the Clustal Co sequences. The data are the mean ± SEM of three cultures per group, and three wells per culture were assayed (*n* = 9/treatment group). An independent samples *t*-test was used to analyze the statistical differences between groups. ^∗^*p* < 0.05; ^∗∗^*p* < 0.01.

It is well-known that that *TP63* gene has multiple transcripts in mammals. However, only one transcript has been identified in chickens; this transcript is short, without a 5′UTR or 3′UTR (NM_204351.1 and AB045224.1). To study the *TP63* transcripts in chickens, we collected total mRNA from chicken embryonic skeletal muscle and pooled tissues, respectively. By using 5′ rapid-amplification of cDNA ends (RACE) and 3′ RACE, we found two *TP63* transcripts existing in chicken tissues (accession number MH238465 and MH238464 in the NCBI database). One of the transcripts which we obtained from skeletal muscle mRNA, has a gene structure similar to that of the *TP63* transcript in NCBI (NM_204351.1), but our transcript contained a 178 bp 5′UTR and a 2,721 bp 3′UTR sequence (**Figure 2A**). The other transcript was obtained from pooled tissues mRNA (**Figure 2A**). This transcript also had a 2,721 bp 3′UTR but the transcription start site was different from that of the first transcript (**Figure 2A**). By using ORFfinder, we obtained several potential ORFs in these two transcript (**Supplementary Figure S1**). The two longest ORFs among the predicted ORFs were then marked and subjected to BLAST analysis to find conserved proteins in the reference proteins database. The BLAST results showed that the ORF predicted from one of the chicken *TP63* transcripts is conserved with TP63 isoform a (also known as TAp63α) in mice, whereas the other chicken *TP63* transcript is conserved with TP63 isoform d (also known as ΔNp63α) in mice (**Supplementary Figure S2**). The BLAST search also showed that the chicken TAp63α and ΔNp63α have high percent identities to the homologous proteins in quail, ducks, humans, mice, rats, pigs, and cattle (**Figures 2B,C**). Amino acid alignment of the TAp63α and ΔNp63α proteins showed that these two proteins were strongly conserved among mammals and birds (**Figures 2D,E**). Therefore, these results suggested that *TAp63*α and Δ*Np63*α are two conserved transcripts expressed in chicken tissues.

### *TAp63*α and Δ*Np63*α Play Opposite Roles in Chicken Myogenic Differentiation

**FIGURE 3:**
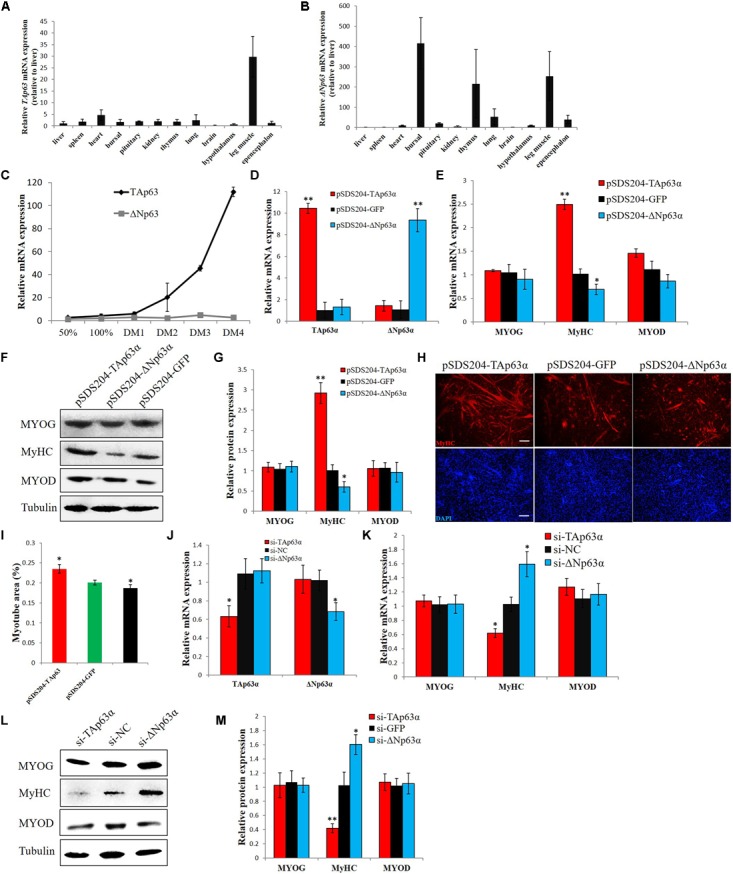
*TAp63*α and Δ*Np63*α play opposite roles in chicken myogenic differentiation. **(A)** qPCR for detecting the relative mRNA expression of *TAp63* in 12 tissues from 7-week-old chickens. **(B)** qPCR for detecting the relative mRNA expression of Δ*Np63* in 12 tissues from 7-week-old chickens. **(C)** Relative mRNA expression of *TAp63* and Δ*Np63* during chicken primary myoblast differentiation. **(D)** Relative mRNA expression of *TAp63* and Δ*Np63* after the transfection of pSDS204-TAp63α, pSDS204-ΔNp63α, or pSDS204-GFP. **(E)** Relative mRNA expression of muscle differentiation marker genes after the transfection of pSDS204-TAp63α, pSDS204-ΔNp63α, or pSDS204-GFP. **(F)** Protein expression of muscle differentiation marker genes after the transfection of pSDS204-TAp63α, pSDS204-ΔNp63α, or pSDS204-GFP. **(G)** Relative protein expression after transfection of pSDS204-TAp63α, pSDS204-ΔNp63α, or pSDS204-GFP. **(H)** MyHC immunostaining in primary myoblasts transfected with pSDS204-TAp63α, pSDS204-ΔNp63α, or pSDS204-GFP and differentiated for 48 h. The nuclei were visualized with DAPI. Bar, 100 μm. **(I)** Myotube area (%) at 48 h after the transfection of pSDS204-TAp63α, pSDS204-ΔNp63α, or pSDS204-GFP. **(J)** Relative mRNA expression of *TAp63* and Δ*Np63* after transfection of si-TAp63α, si-ΔNp63α, or si-NC. **(K)** Relative mRNA expression of muscle differentiation marker genes after transfection of si-TAp63α, si-ΔNp63α, or si-NC. **(L)** Western blotting results for muscle differentiation marker genes after the transfection of si-TAp63α, si-ΔNp63α, or si-NC. **(M)** Relative protein expression after the transfection of si-TAp63α, si-ΔNp63α, or si-NC. The data are the mean ± SEM of three cultures per group, and three wells per culture were assayed (*n* = 9/treatment group). An independent samples *t*-test was used to analyze the statistical differences between groups. ^∗^*p* < 0.05; ^∗∗^*p* < 0.01.

Next, we studied the expression of *TAp63*α and Δ*Np63*α in chicken tissues. Using TA- and ΔN-specific primers and a real-time polymerase chain reaction (qPCR) assay, we found that *TAp63* has higher expression in skeletal muscle than in other tissues (**Figure 3A**), whereas Δ*Np63* has high expression in bursal and thymus tissue and in skeletal muscle (**Figure 3B**). During myogenic differentiation, the expression of *TAp63* was gradually upregulated, whereas the expression of Δ*Np63* was relatively stable (**Figure 3C**). As **Figures 1D,E** show, our siRNA designed for *TP63* was not isoform-specific (**Supplementary Figure S3**). To further study the functions of *TAp63*α and Δ*Np63*α in chickens, we constructed *TAp63*α and Δ*Np63*α overexpression vectors. Transfecting one of the TP63 overexpression vectors would upregulate the expression of that transcript without affecting the expression of the other transcript (**Figure 3D**). We then transfected these two vectors into chicken primary myoblasts, and induced the cells to differentiate. After 48 h, we found that *TAp63*α overexpression upregulated the mRNA and protein expression of *MyHC* (**Figures 3E–G**), which is a terminal marker of myogenic differentiation. However, Δ*Np63*α overexpression repressed *MyHC* expression (**Figures 3E–G**). Additionally, MyHC immunofluorescence showed that *TAp63*α and Δ*Np63*α have opposite effects on myotube formation (**Figure 3H**), as indicated by the quantification of myotube areas (**Figure 3I**). On the other hand, we used isoform-specific siRNAs to knockdown the expression of *TAp63*α and Δ*Np63*α (**Figure 3J**). *TAp63*α knockdown downregulated *MyHC* mRNA and protein expression, whereas Δ*Np63*α knockdown upregulated *MyHC* mRNA and protein expression (**Figures 3K–M**). Altogether, these results indicated that *TAp63*α and Δ*Np63*α play opposite roles in chicken myogenic differentiation.

### *TAp63*α and Δ*Np63*α Regulate Different Sets of Genes in Myoblasts

**FIGURE 4:**
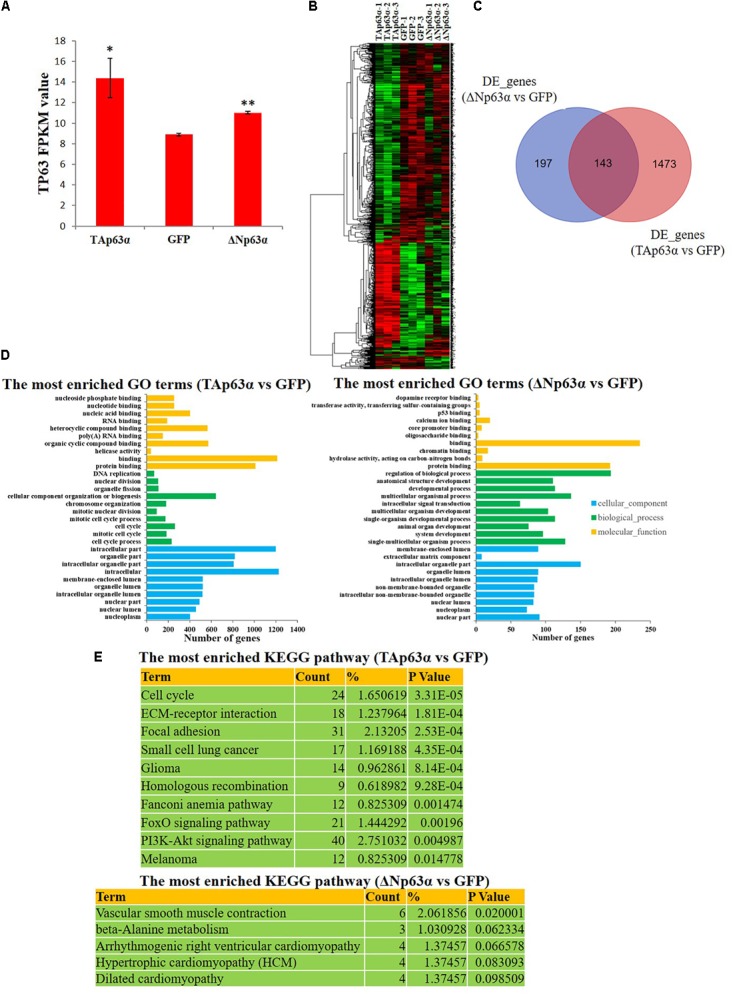
*TAp63*α and Δ*Np63*α regulate different sets of genes in myoblasts. **(A)** RNA-seq result showed the success of *TAp63*α and Δ*Np63*α overexpression in chicken primary myoblast. **(B)** Hierarchical clustering of differential expressed genes (DEGs) between *TAp63*α (TP), Δ*Np63*α (NP) and GFP (NC) overexpression groups. **(C)** The Venn diagram of DEGs between “ΔNp63α vs. NC” and “TAp63α vs. NC.” **(D)** GO enrichment for the DEGs between “TAp63α vs. NC” and “ΔNp63α vs. NC.” The *y*-axis represents GO terms and the *x*-axis represents number of genes (with *p*-value <0.05). **(E)** The most enriched KEGG pathway for the DEGs between “TAp63α vs. NC” and “ΔNp63α vs. NC.”

To study the downstream genes of *TAp63*α and Δ*Np63*α in chicken myoblast, we overexpressed these two transcripts in chicken primary myoblasts and collected the mRNA for RNA sequencing (RNA-seq). The RNA-seq results showed the successful overexpression of *TAp63*α and Δ*Np63*α in myoblasts (**Figure 4A**), and numerous differentially expressed genes (DEGs) between the groups (**Figure 4B** and **Supplementary Table S2**). From the gene expression heatmap we can see that the DEGs induced by *TAp63*α and Δ*Np63*α are very different (**Figure 4B**). *TAp63*α overexpression resulted in 1616 significantly DEGs, whereas Δ*Np63*α overexpression resulted in only 340 significantly DEGs (**Figure 4C**); furthermore, there were only 143 overlapping DEGs between these two groups (**Figure 4C**). GO analysis revealed that the *TAp63*α-induced DEGs are enriched in the cell cycle, DNA replication, and nucleotide binding terms (**Figure 4D**), whereas Δ*Np63*α-induced DEGs are enriched in the developmental process, regulation of biological process, and protein binding terms (**Figure 4D**). In addition, KEGG pathway analysis revealed that *TAp63*α-induced DEGs are enriched in the cell cycle pathway, whereas Δ*Np63*α-induced DEGs are enriched in the muscle development- or myopathy-related pathways (**Figure 4E**). Therefore, these results indicated that *TAp63*α and Δ*Np63*α regulate different set of genes in myoblasts.

### Identification of Major Downstream Regulatory Pathways and Functional Gene Groups of *TAp63*α and Δ*Np63*α in Myoblast

**FIGURE 5:**
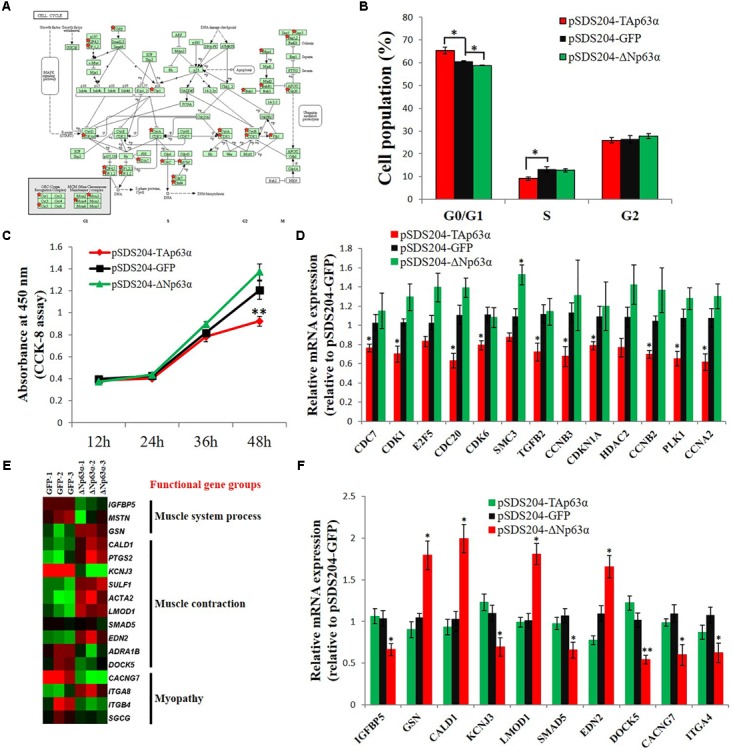
Identification of major downstream regulatory pathways and functional gene groups of *TAp63*α and Δ*Np63*α in myoblasts. **(A)** DEGs between TAp63α overexpression myoblast and NC myoblast were enriched in the cell cycle pathway. Red stars indicate the genes that are differentially expressed between TAp63α and NC group. **(B)** Myoblasts were transfected with pSDS204-TAp63α, pSDS204-ΔNp63α, or pSDS204-GFP, followed by 48 h culture, and the cell cycle phase was then analyzed. **(C)** CCK-8 assay was performed to access the effects of *TAp63*α and Δ*Np63*α overexpression on myoblast proliferation. **(D)** qPCR validation of the DEGs enriched in cell cycle pathway. **(E)** The heat-map of genes related to muscle system process, muscle contraction, and myopathy. **(F)** qPCR validation of the DEGs related to muscle system process, muscle contraction, and myopathy. The data are the mean ± SEM of three cultures per group, and three wells per culture were assayed (*n* = 9/treatment group). An independent samples *t*-test was used to analyze the statistical differences between groups. ^∗^*p* < 0.05; ^∗∗^*p* < 0.01.

From the GO and KEGG pathway analysis results, we can see that the cell cycle is a potential target pathway of *TAp63*α. Many genes involved in the cell cycle pathway were differentially expressed in *TAp63*α-overexpressing myoblasts compared to control myoblasts (**Figure 5A**). Notably, *TAp63*α overexpression increased the number of cells in the G0/G1 stage and decreased the number of cells in the S stage (**Figure 5B**), whereas Δ*Np63*α overexpression decreased the number of cells in the G0/G1 stage (**Figure 5B**). The CCK-8 assay showed that *TAp63*α overexpression inhibited cell proliferation, whereas Δ*Np63*α overexpression had no significant effect on this process (**Figure 5C**). The qPCR results verified that the expression of many DEGs in the cell cycle pathway was significantly inhibited in *TAp63*α-overexpressing myoblasts but not in Δ*Np63*α-overexpressing cells (**Figure 5D**).

From the GO and KEGG pathway analysis results, we found that many of the Δ*Np63*α downstream genes were involved in the muscle system process, muscle contraction, and myopathy (**Figure 5E**). Our qPCR results validated that Δ*Np63*α can regulate the expression of these genes (**Figure 5F**). Therefore, these results suggested that the cell cycle is a potential regulatory pathway targeted by *TAp63*α in myoblasts and that genes involved in muscle system process, muscle contraction, and myopathy were potential downstream targets of Δ*Np63*α in myoblasts.

## Discussion

**FIGURE 6:**
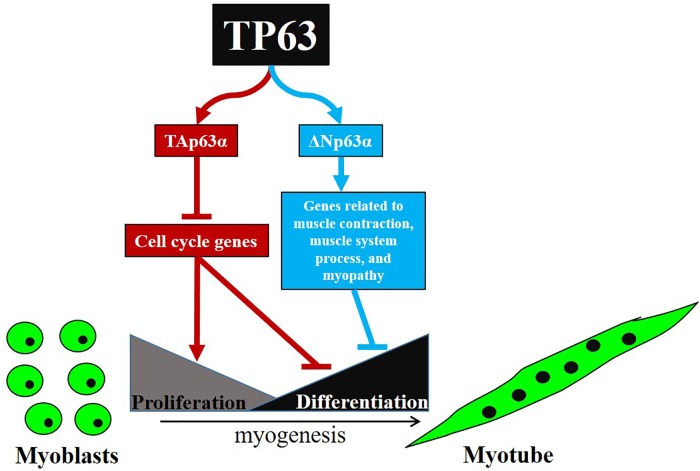
Schematic illustration for signaling pathways of myoblast proliferation and differentiation regulated by *TP63* gene.

In this study, we cloned the full-length cDNA of the chicken Δ*Np63*α, and found the full-length *TAp63*α transcript, which has never been reported in chickens. *TAp63*α and Δ*Np63*α have different expression patterns and perform different functions during myoblast differentiation. *TAp63*α inhibits myoblast proliferation and promotes myoblast differentiation by regulating cell cycle-related genes, whereas Δ*Np63*α inhibits myoblast differentiation by regulating genes related to muscle contraction, muscle system process, and myopathy (**Figure 6**).

The *TP63* gene has at least ten transcripts in humans, such as *TAp63*α, *TAp63*β, *TAp63*γ, *TAp63*δ, *TAp63*ε, Δ*Np63*α, Δ*Np63*β, Δ*Np63*γ, Δ*Np63*δ, and Δ*Np63*ε (Mangiulli et al., 2009). *TA* and Δ*N* represent 5′ variants, and **α, **β, **γ, **δ, and **ε represent 3′ variants. However, we found only *TAp63*α and Δ*Np63*α in chicken tissues. The 5′RACE result identified the *TA* and Δ*N* transcripts, whereas 3′RACE identified only the **α transcript. The other *TP63* transcripts may also exist in chickens, but these transcripts may be expressed in different tissues with different time-course expression profiles. Because it is hard to design primers that can identify every single transcript, we used *TA*- and Δ*N*-specific primers to detect *TAp63* and Δ*Np63* in chicken tissues. Notably, *TAp63* is mainly expressed in skeletal muscle, whereas Δ*Np63* can be expressed in multiple tissues. The upregulation of *TAp63* and the stable expression of Δ*Np63* during chicken myoblast differentiation were consistent with the results in C2C7 (Cefalu et al., 2015). However, the isoform-specific expression of the *TP63* transcripts during myoblast differentiation needs further investigation.

The *TP63* transcripts encode the corresponding isoforms and play different roles in cellular processes. We found that *TAp63*α and Δ*Np63*α are not only differentially expressed but also play opposite roles in myogenic differentiation. Similarly, protein kinase C isoforms can play opposite roles in the proliferation, differentiation, and apoptosis of human HaCaT keratinocytes (Papp et al., 2004), and p38 isoforms exert opposite effects on MKK6-mediated VDR transactivation (Pramanik et al., 2003). These phenomena indicate that one gene can perform at least two different functions by expressing different isoforms during a single cellular process. However, the expression of these isoforms would be strictly controlled by gene expression regulation programs, such as alternative promoters, alternative splicing, and post-translational processing, so that the appropriate functional isoform is expressed at the appropriate time. In addition to playing opposite roles during a single cellular process, one TP63 isoform may influence the function of another during myogenic differentiation. For example, the upregulation of ΔNp63α inhibited myoblast differentiation, which was induced by TAp63α. A previous study showed that ΔNp63 can directly compete for TAp63 target promoters or sequester TAp63 to form inactive tetramers (Candi et al., 2007). Therefore, it is possible that the two isoforms compete for a sub-set of target genes during myogenic differentiation. In this case, identifying the target genes of these two isoforms is important in order to reveal the mechanism of action of *TAp63*α and Δ*Np63*α, as well as to confirm the interaction between these two isoforms.

*TP63* is a well-known tumor suppressor gene that can regulate cell cycle progress and inhibit cancer cell proliferation (Benard et al., 2003). Here, we found that the *TAp63*α isoform is capable of inducing cell cycle arrest in myoblasts and is able to inhibit myoblast proliferation. Cell cycle arrest is important for myogenic differentiation. Myoblasts permanently exit from the cell cycle during terminal differentiation (Derer et al., 2002). The upregulation of *TAp63*α during myoblast differentiation may promote cell cycle arrest, therefore, facilitating the terminal differentiation of myoblasts. In addition, TP63 has been reported to play roles in the late stage of myogenic differentiation (Cefalu et al., 2015). The knockdown of TAp63gamma would affect the expression of genes related to myogenesis and skeletal muscle contractility (Cefalu et al., 2015). Our results also showed that TP63 isoforms could regulate myogenesis and muscle contraction and that the expression of many myogenic differentiation genes, such as *MYH9*, *MYH10*, *RUNX1*, *ROCK1*, *ROCK5*, *MSTN*, *SMAD5*, and *CDKN1A*, were significantly changed (**Supplementary Table S2**). Therefore, the *TP63* gene is involved in skeletal muscle cell proliferation and differentiation.

TP63 is a conserved transcription factor with multiple binding sites in the genome (McDade et al., 2014). TP63 regulates the expression of downstream genes by through directly affecting the transcription of genes to whose promoter it binds (McDade et al., 2012). A better way to identify downstream genes of TP63 is via chromatin immunoprecipitation (ChIP)-related assays, such as ChIP-chip or ChIP-sequencing. However, there is no isoform-specific ChIP-grade antibody for TP63 in chickens. Furthermore, studies investigating the genome-wide binding of TP63 did not use isoform-specific antibodies (McDade et al., 2012, 2014). Not only will structural variations of protein isoforms affect protein function in cellular processes, but the binding sites will also be different (Kiselev et al., 2012). Therefore, it is important to develop isoform-specific antibodies for TP63 to better understand its genome-wide regulation in specific cell types. In this study, we used isoform-specific overexpression assays and identified a list of *TAp63*α- and Δ*Np63*α-specific potential downstream genes in myoblasts. Previous studies on TP63 in myogenesis used an siRNA strategy for functional investigation (Cam et al., 2006; Cefalu et al., 2015). The siRNAs designed to knockdown *TP63* expression were not isoform-specific (Cam et al., 2006; Cefalu et al., 2015); therefore, it is hard to demonstrate the specific function of each TP63 isoforms in myogenesis. Here, we used an isoform-specific overexpression assay to investigate the function of *TAp63*α and Δ*Np63*α in myoblast proliferation and differentiation. Although this strategy is not optimal for screening the downstream target genes of *TAp63*α and Δ*Np63*α, our results identified the specific functions of these two isoforms in myoblast differentiation. In conclusion, *TP63* is important for skeletal muscle development, and the isoforms of TP63, namely, TAp63α and ΔNp63α, play opposite roles in myoblast differentiation.

## Author Contributions

WL, QN, and XZ designed the experiments. WL and XZ wrote the manuscript. WL, XR, JC, LL, SL, and TC did the experiments.

## Conflict of Interest Statement

The authors declare that the research was conducted in the absence of any commercial or financial relationships that could be construed as a potential conflict of interest. The reviewer DLC and handling Editor declared their shared affiliation.

## Footnotes

1 http://www.ncbi.nlm.nih.gov/geo

## Abbreviations

DF-1: chicken embryo fibroblast cell line
DM: differentiation medium
GM: growth medium
GO: Gene Ontology
KEGG: Kyoto Encyclopedia of Genes and Genomes
miRNA: microRNA
NC: negative control
qPCR: quantitative polymerase chain reaction
TP63: tumor protein 63
UTR: untranslated region
w: week.
