# Supplementary material for: TP63 Transcripts Play Opposite Roles in Chicken Skeletal Muscle Differentiation
Source: Front Physiol. 2018 Sep 18;9:1298. doi: 10.3389/fphys.2018.01298 (PMC6157316; doi:10.3389/fphys.2018.01298)
Supplement: Supplementary file 3 [file Data_Sheet_3.DOCX]

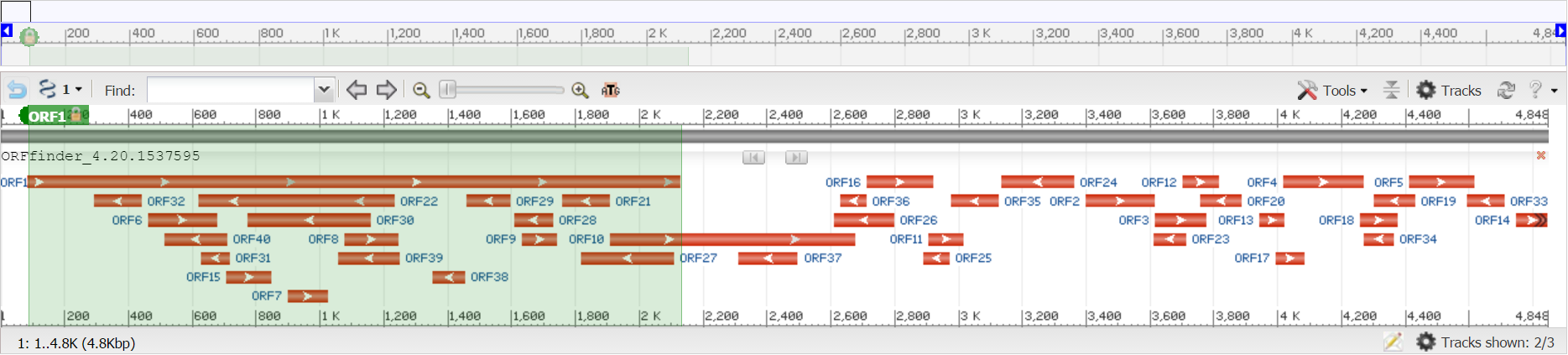


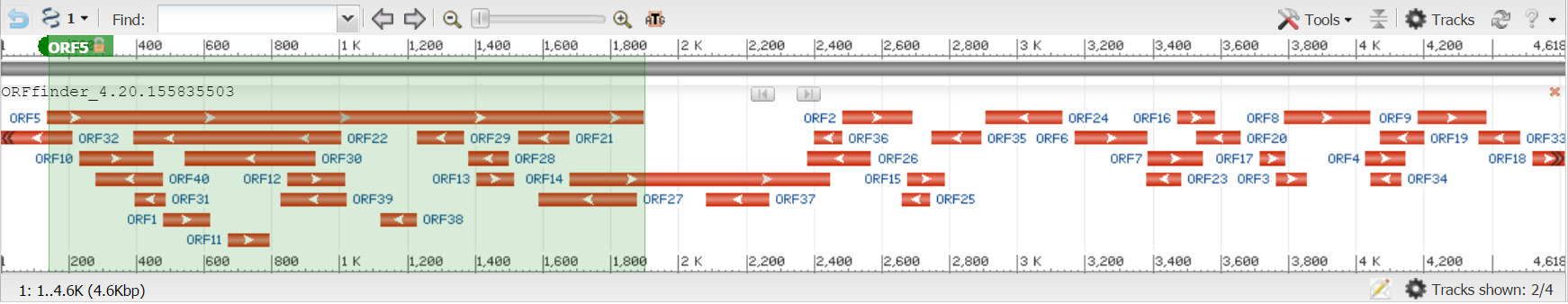


Figure S1: The predicted ORFs of TAp63α (upper) and ΔNp63α (lower) by using ORFfinder.


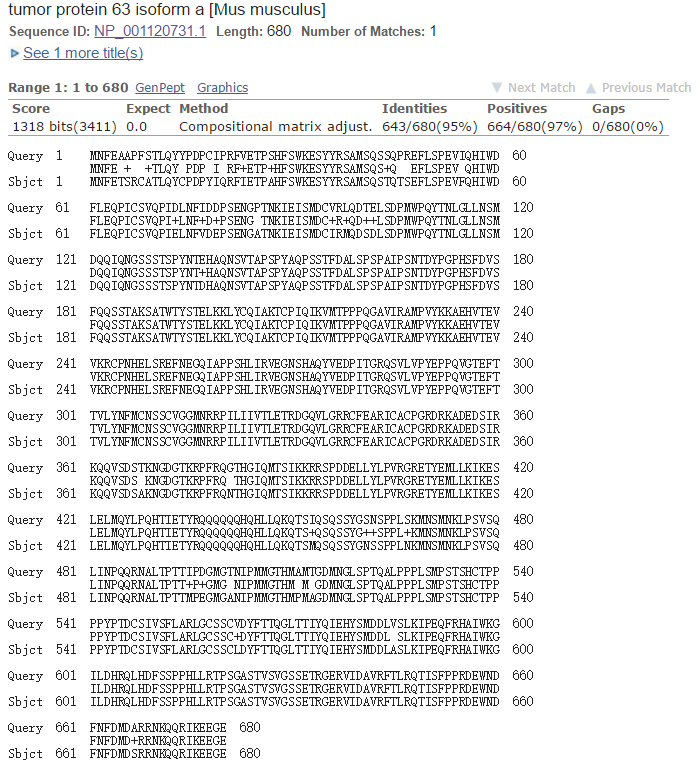

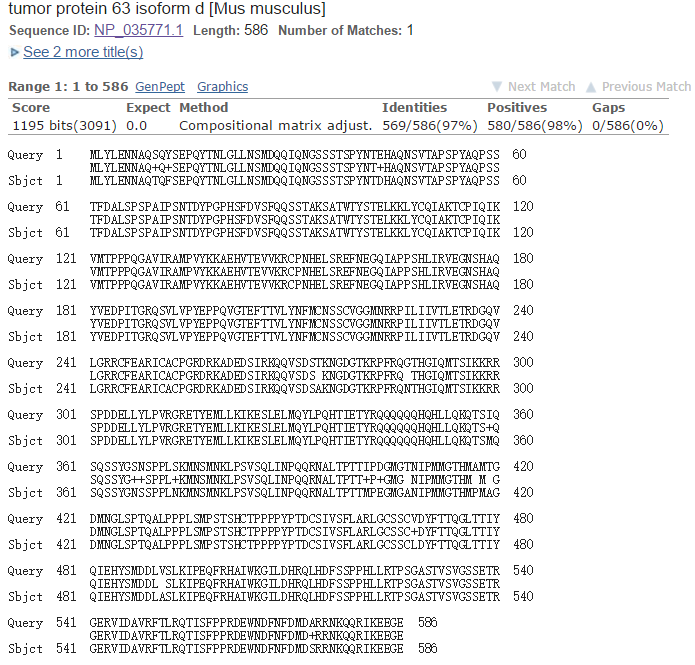


Figure S2: Left: BLAST results between chicken TAp63α and mouse TP63 isoform a; Right: BLAST results between chicken ΔNp63α and mouse TP63 isoform d.


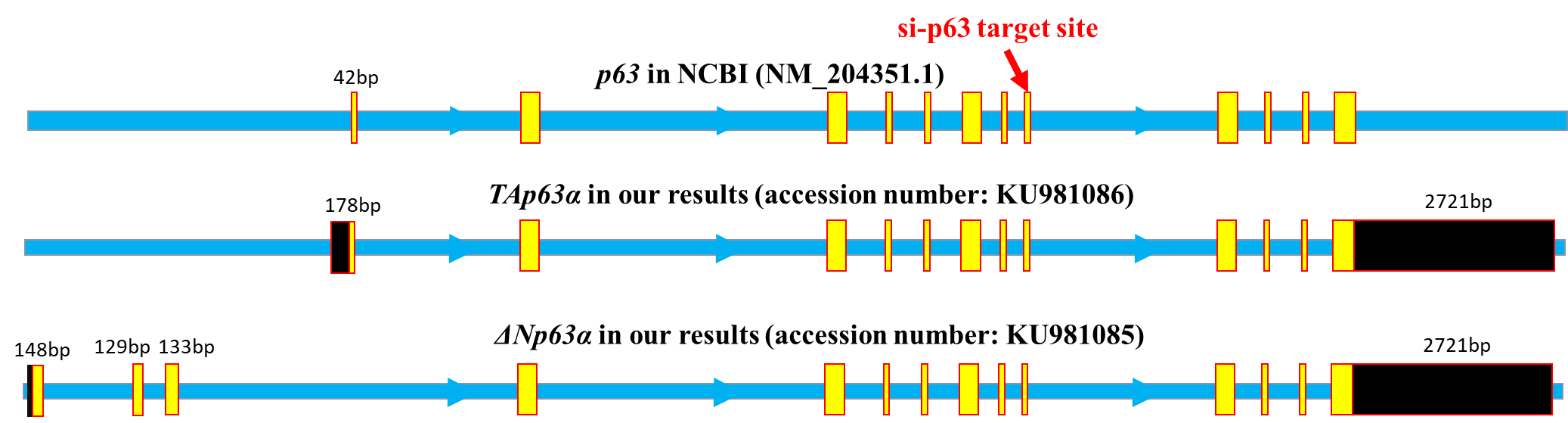


Figure S3: Target site of si-p63 used in this study.
